# Reliability, validity and minimal detectable change of the Chinese Version of the Assessment of Physical Activity in Frail Older People (APAFOP-C)

**DOI:** 10.1186/s12877-024-05167-y

**Published:** 2024-07-06

**Authors:** Yuelin Li, Linyu Lyu, Xing Fan, Lijuan Xu, Yan Li, Rhayun Song

**Affiliations:** 1 College of Nursing, Chungnam National University, Daejeon, Republic of Korea; 2 School of Medicine, Lishui University, Lishui, China; 3 Department of Education, Chungnam National University, Daejeon, Republic of Korea

**Keywords:** Activities of daily living, Exercise, Validation study, Frail older adults

## Abstract

**Background:**

Physical activity (PA) is essential in mitigating frailty syndrome, and it is necessary to measure PA in older adults with frailty. Assessment of Physical Activity in Frail Older People (APAFOP) is a suitable patient-reported outcome measure (PROM) for assessing PA among older adults with frailty. This study aimed to determine the reliability, validity and minimal detectable change of the Chinese version of the APAFOP (APAFOP-C).

**Methods:**

This cross-sectional validation study was designed to measure the reliability and criterion validity of the APAFOP-C with 124 frail community-residing older adults. APAFOP-C was completed twice within an interval of 7–17 days to determine test-retest reliability. The investigator triangulation method was used to investigate inter-rater reliability, and a pedometer was used as the reference measurement to assess the criterion validity. Reliability and criterion validity were assessed using the intraclass correlation coefficient (ICC_2,1_), Pearson correlation coefficient for normally distributed variables, Spearman correlation coefficient, Wilcoxon signed-rank test for skewed variables, and the minimal detectable change at 95% level of confidence (MDC_95_). Agreement assessment was conducted using Bland-Altman plots for inter-rater reliability and criterion validity. Kendall’s W test assessed absolute agreement among three raters in inter-rater reliability. The Mann-Whitney U test was used to evaluate whether any particular day was more representative of certain daily activities.

**Results:**

Total PA on any arbitrarily chosen day illustrates daily activity (*Z*= -0.84, *p* = 0.40). The APAFOP-C exhibited strong-to-very strong test-retest reliability (ICC_2,1_=0.73–0.97; Spearman ρ = 0.67–0.89), and the total PA score demonstrated MDC_95_ < 10%. Inter-rater reliability was also strong-to-very strong (ICC_2,1_=0.96–0.98; Spearman *ρ* = 0.88–1.00), and moderate criterion validity when compared with total PA score on pedometer readings (Spearman ρ = 0.61). Limits of agreement among different raters regarding the APAFOP-C and the pedometer were narrow.

**Conclusion:**

The APAFOP-C was found to have limited but acceptable psychometric properties for measuring PA among community-dwelling older adults with frailty in China. It was a feasible comparative PROM for assessing PA worldwide. Practitioners can develop individualized exercise programs for frail older adults and efficiently track changes in PA utilizing the APAFOP-C.

**Supplementary Information:**

The online version contains supplementary material available at 10.1186/s12877-024-05167-y.

## Introduction

Once a person reaches around 70 years old, a new phenotype of a transitional and multidimensional condition distinct from any single chronic disease emerges and develops as a normal part of the aging process, which leads to a progressive decline in physiological functional status, known as frailty [1]. Low-intensity physical activity (PA) and four other clinical syndromes form the frailty phenotype: unintentional weight loss, self-reported exhaustion, weak grip strength, and slow walking speed [2].

Research has confirmed that PA preserves and improves the function of many physiological systems that are operating abnormally in older adults with frailty, such as sarcopenia [3], protein synthesis [4], inflammation [5, 6], and anemia [7]. There is also increasing evidence that older adults with frailty who maintain a physically active lifestyle benefit from improved physical characteristics such as physical endurance, physical performance, and functional status [8–10]. However, given that “lack of time and interest,” “health status,” and “fear” are the most commonly reported obstacles to PA in the oldest old adults [11], studies have also found that even low-dose PA resulted in significant linear reductions in frailty [12] and all-cause mortality [13].

Methods for assessing PA include objective methods (e.g., accelerometry, pedometer, and doubly labeled water) and subjective methods (e.g., PA questionnaires and activity logs) [14]. Objective measurements allow for detailed accounts of PA intensity and frequency but are highly time-consuming and burdensome the assessor when performing large-scale epidemiological studies [15]. In contrast, subjective measures are an excellent method for identifying the dimensions of PA, which includes frequency, type, intensity, and time and, in some instances, estimating the amount of metabolic equivalents (MET) and energy expenditure level [16]. Moreover, self-reported data quantification enables a practical and low-cost option to capture data at the population level [17].

Some studies have used several subjective measurements to measure populations of older adults. Since these measures were not specifically designed for older adults with frailty, they cannot effectively capture intermittent, sporadic, unstructured PA with short stochastic bursts, or non-exercise activity thermogenesis. It is worth noting that these unique characteristics of PA are its dominant components in older adults with frailty [18], and failure to capture them would lead to under- or over-estimation of PA among older adults with frailty [19, 20], which would result in unreliable measurement results [21, 22]. The Assessment of Physical Activity in Frail Older People (APAFOP) has been demonstrated to be a feasible and pragmatic patient-reported outcome measure (PROM) with high utility in research and for capturing PA performed by older adults with frailty and institutionalized older adults [23, 24]. Furthermore, to promote widespread international use and cater to cultural differences and specific populations, our research team used a systematic cross-cultural adaptation process established by Beaton and colleagues in 1994 and a rigorous cognitive interviewing method to translate and cross-culturally adapt the APAFOP to the Chinese context [25, 26]. The translation and cross-cultural adaptation processes of the Chinese version of the APAFOP (APAFOP-C) were conducted and reported in a previous study [27].

The purpose of the present study was to determine the reliability, validity and minimal detectable change of the APAFOP-C among community-residing frail older adults in China. Specifically, it aimed to determine the criterion validity of the APAFOP-C compared with a pedometer as a standardized tool to assess physical activity and to evaluate the reproducibility (inter-rater and test-retest reliability) of the APAFOP-C.

## Method

### Study design

This validation study utilized a cross-sectional survey to evaluate the reliability and criterion validity of the APAFOP-C.

### Setting and sample

**Fig. 1:**
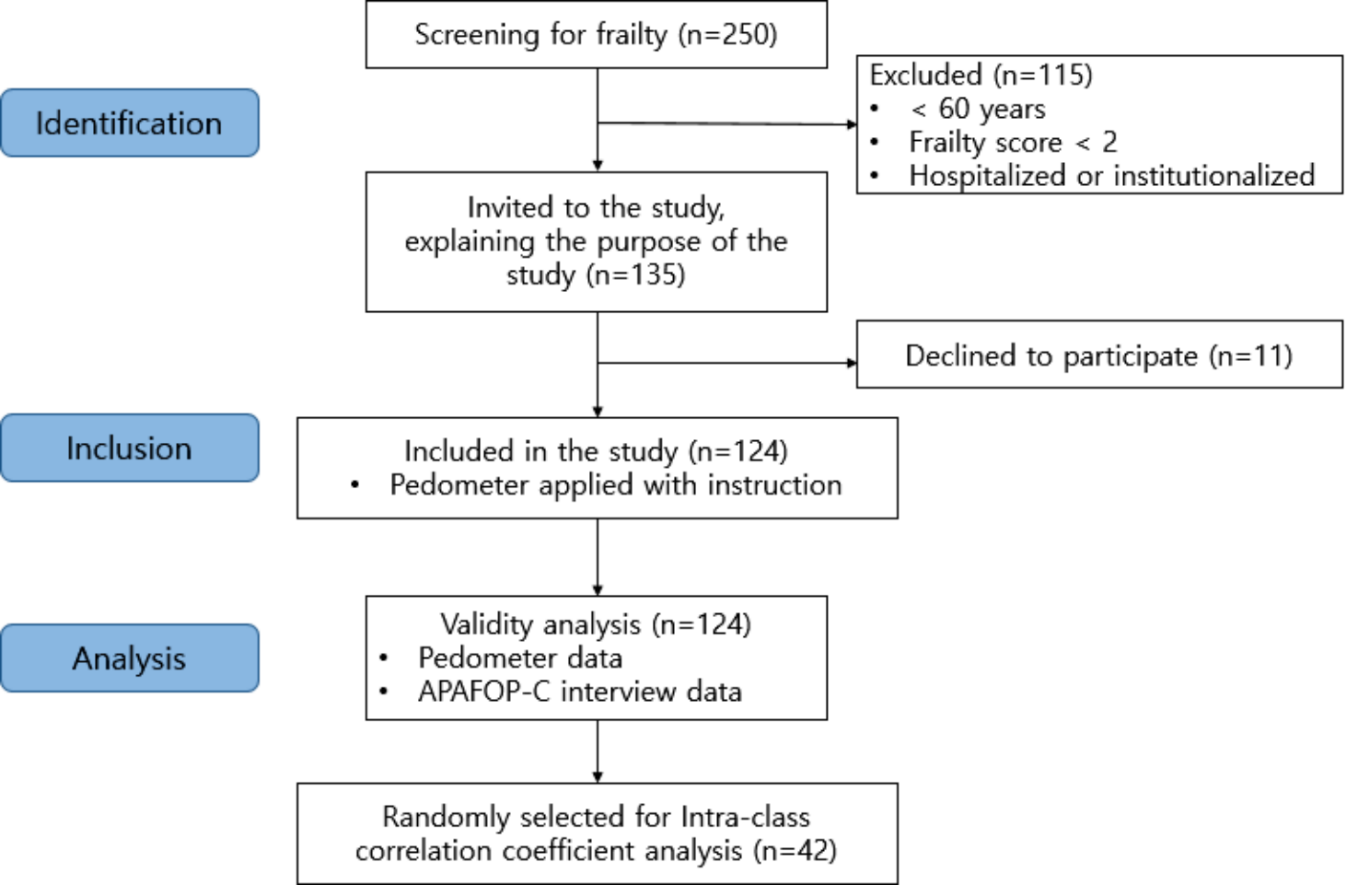
Flow of the study process

A convenience sampling method was used to recruit older adults with frailty who live in the northeastern part of China by placing flyers on public advertisement boards and by word-of-mouth. Those aged 60 and older, residing in the community, and scoring two or more on the Chinese FRAIL scale [28] were eligible for inclusion. We selected this criterion according to the Chinese FRAIL scale [28], indicating robustness at 0 points, pre-frailty at 1 point, and frailty at 2 points or higher. The study excluded older adults who were institutionalized or hospitalized, as well as those who were incapable of communicating or responding during the interview. A total of 124 frail older adults living in the community were included in the study based on the inclusion criteria, and they were instructed on how to use pedometers to assess their daily activities. Among 124 participants, we randomly selected 42 older adults to assess test-retest reliability (Fig. 1). In this study, the sample size for intraclass correlation coefficient (ICC) was calculated with a correlation coefficient of 0.7 as an effect size [23], power 0.8, and target width 0.3 of the 95% confidence interval of ICCs (ICC_2,1_). The required same size was a minimum of 40 for 3 raters with systematic deviation based on the recommendation by Mokkink et al. [29].

### Data collection

Data collection was performed from January 12 to July 3, 2022, using an interviewer-administered questionnaire and objective tools. Each participant was invited to a face-to-face interview on day 0 (D0), and a research assistant fully explained the purpose of the study. Participants were asked to complete a sociodemographic questionnaire and undergo anthropometric measurements after providing informed consent. To ensure participant engagement, each individual was provided with a pedometer and a gift valued between 5 and 10 Chinese Yuan.

Sociodemographic data such as age, sex, marital status, education level, retirement status, and perceived health were collected. Anthropometric data, including height and weight, were collected from each participant to calculate body mass index (BMI). In this study, participants were classified by BMI level: underweight (< 18.5 kg/m^2^), normal weight (18.5–24.9 kg/m^2^), overweight (25.0–29.9 kg/m^2^), and obese (> 30 kg/m^2^).

Inter-rater reliability was assessed using an investigator triangulation method [30]. Three raters were invited, including one who was involved in the manual’s development as a reference (reference-rater 1). The second rater (rater 2) did not receive any training but read the user manual and familiarized himself with the questionnaire in advance. A third rater (rater 3) was given formal training on how to administer the questionnaire and avoid systematic errors during the data collection process. Reference-rater 1 recorded interviews and sent the recordings to the other two raters for independent scoring in order to minimize the potential for bias in inter-rater reliability. Based on the data independently scored by each rater, we evaluated whether training or a user manual could reduce systematic errors when applying the APAFOP-C.

### Objective assessment of PA

A pedometer (Yamax SW-200, Yamax, Tokyo, Japan) was used to measure the PA of the participants. The device contains a motion sensor that captures and records motion and responds to vertical acceleration of the human body, enabling the direct comparison of patient reports on the APAFOP-C for both individual domains and the total score. Several studies have previously demonstrated its validity, reliability, and accuracy, as well as its superior performance under both free-living [31, 32] and controlled laboratory conditions [33, 34]. In addition, the Yamax pedometer is commonly used in applied research in older populations [31, 35, 36].

Each participant was provided with a pedometer along with verbal and written instructions on how to operate it. Each participant wore a pedometer on the waistband of their thigh, and any movement above a threshold was recorded as a completed step. A pedometer was also given to participants to reset to zero when they awoke the following morning (D1) after the baseline measurement, and participants were instructed to wear it throughout the day except while sleeping or bathing, and to continue their regular physical activity routines during the investigation. Steps taken during the day until going to bed were recorded by participants. The research assistant met with participants again on the second experimental day (D2) to collect the pedometer data and asked the participants to recall their PA from the previous day by administering the APAFOP-C. Finally, 7–14 days after D1 [37, 38], 42 randomly selected participants completed the APAFOP-C again to determine test-retest reliability.

### Subjective assessment of PA

The APAFOP was initially developed to assess PA frequency and duration among older and frail populations in six domains: walking, outdoor activity, indoor activity, sitting, lying down, and sports activity. PA intensity was rated on the APAFOP according to a MET-based scoring system, and the developers adjusted the MET value according to the PA characteristics of this population. The adjusted MET value ranged from 1 to 4 depending on the PA sub-domain, including low-intensity daily activities or recreation-level to high-intensity sports. The score for each domain was calculated by multiplying the MET levels of activities and duration of the respective activity over a day, and the total PA score was calculated by summing the contribution of six domains. Higher scores indicated higher PA levels.

The APAFOP was translated into Chinese and cross-culturally adapted following strict and systematic guidelines [25, 26, 39]. In the APAFOP-C, the intensity and scoring method remained the same to ensure international comparability. However, the questionnaire layout was adjusted to increase the convenience for interviewers, and some PA items were changed or expanded to adapt it to the Chinese context. A previous study demonstrated that the APAFOP-C obtained good content validity and was considered comprehensive and relevant in assessing the PA of older adults with chronic conditions, various levels of limited physical and cognitive function, and sedentary behavior in China [27] (see related files).

### Data analysis

All data were entered into Excel, and statistical analyses were performed using SPSS software (version 26.0, SPSS, Chicago, IL, USA). Self-reported PA data were scored according to the APAFOP-C user manual. Variables in this study were reported as numbers and percentages or as mean ± SD, while medians and inter-quartile ranges (IQRs) were used for the variables with skewed distributions. The Mann-Whitney U test was used to evaluate whether an arbitrarily chosen day was representative of certain daily activities. The Kolmogorov-Smirnov test was performed to assess normality. Moreover, results in this study were not stratified by sex since the difference in baseline was not detected in both pedometer readings and all PA-related variables based on the Mann-Whitney U tests.

Reproducibility of the APAFOP-C was assessed by test-retest reliability and agreement among three raters. Test-retest reliability was assessed by comparing scores of the APAFOP-C performed on D1 and at intervals of 7–14 days [38] using reliability coefficient (ICC_2,1_). In addition, a standard error of measurement (SEM) and minimal detectable change at the 95% confidence level (MDC_95_) for absolute reliability were provided following the equation: $SEM=SD\times \surd (1-ICC)$ and $MDC=SEM\times \sqrt{2}\times 1.96$. Inter-rater reliability among three raters with the rater 1 as a reference-rater was evaluated using Kendall’s W test and ICC_2,1_. Bland-Altman plots were created for inter-rater reliability based on the mean values between each pair of raters (reference-rater1 - rater2; reference-rater1 - rater3; rater2 - rater3).

The criterion validity of APAFOP-C was determined by comparing PA scores (total, intensity-based, and each subdomain) with total steps measured by pedometers. We categorized intensity-based PA into inactive (summated scores for sitting and lying down) and active (summated scores for walking, outdoor activities, indoor activities, and sports). A previous study demonstrated that the correlation between two measures provides information about the strength of the relationship but does not reflect the agreement. Additionally, moderate to high agreement between measures can justify selecting one over another [1, 40]. The Limit of Agreement (LoA) was assessed using Bland-Altman plots with Z-score normalized data for criterion validity. Pearson correlation coefficient was calculated for normally distributed data, while the Wilcoxon signed-rank test and Spearman correlation coefficient were used for non-normally distributed data. We interpreted Pearson correlation coefficients, interclass correlation coefficients, and Spearman correlation coefficients as follows: a correlation coefficient of less than 0.10 is negligible, 0.10–0.39 is weak, 0.40–0.69 is moderate, 0.70–0.89 is strong, and 0.90-1.0 is very strong [41]. The level of statistical significance was set at 0.05 [41].

## Results

### Sociodemographic characteristics of the participants

The study sample comprised 124 older adults with frailty, aged 77.05 ± 5.90 years, ranging from 63 to 88 years; 66 (53.2%) of the participants were male. Among these participants, 54.8% were married or living with a partner, 58.9% had received primary-school education, 61.3% were retired, and 75% self-reported a fair health status. Regarding body mass, 72.6% had a normal BMI. Notably, 17 participants (13.7%) reported that their PA on the investigation day was different from their typical daily patterns. Despite these variations, a Mann-Whitney U test revealed no statistically significant difference in the total PA scores measured on the APAFOP-C among those who performed PA differently (*Z*= -0.84, *p* = 0.40). The median time to complete the checklist (excluding the time to explain the items) per trial was 6.23 min (range 1.12–18.21 min). The total scores of APAFOP-C were 28.59 on average, ranged from 24.5 to 44.5, while their total steps of pedometer reading were 5173, with the range of 466 to 14,665. When categorizing the daily steps of the participants based on the normative data for special population (older adults with chronic health conditions) [42], 83.1% of the participants fell into the normative range, with 5.6% walking less and 11.3% walking more than normative range (Table 1).

**Table 1:** Sociodemographic and anthropometric characteristics *N* = 124

Characteristic	Value
**Age, years**	77.05 ± 5.90 (range 63–88)
**Gender**	
Men	66 (53.2)
Women	58 (46.8)
**Marital status**	
Married	68 (54.8)
Single	43 (34.7)
Separated	6 (4.8)
Widowed	7 (5.6)
**Educational level**	
Primary school or lower	73 (58.9)
Middle school	41 (33.1)
High school or higher	10 (8)
**Retired status**	
Retired	76 (61.3)
Employed	36 (29.0)
Self-employed	5 (4.0)
Caregiver of family member	7 (5.6)
**Perceived health**	
Poor	13 (10.5)
Fair	93 (75)
Good	18 (14.5)
**Activity change compared with usual days** ^**a**^	
More activity	14 (11.3)
As usual	107 (86.3)
Less activity	3 (2.4)
**Chinese FRAIL scale** ^**b**^	
Score = 2	43 (34.7)
Score = 3	77 (62.1)
Score = 4	4 (3.2)
**BMI, kg/m** ^**2**^	23.89 ± 2.68
**BMI classes, kg/m** ^**2**^	
Underweight, < 18.5	1 (0.8)
Normal, 18.5–24.9	90 (72.6)
Overweight, 25.0–29.9	27 (21.8)
Obese, > 30	6 (4.8)
**APAFOP-C total score**	28.59 (range 24.5–44.5)
**Pedometer readings (steps/day)**	5173 (range 466-14665)
**Steps/day level** ^**c**^	
Less	7 (5.6)
Normative (1200–8800)	103 (83.1)
More	14 (11.3)

Data are presented as Mean ± SD, n (%), Median (range); BMI: body mass index;

^a^Chinese FRAIL scores 2 or higher points based on frailty criteria

^b^Mann-Whitney U test *Z*= -0.84, *p* = 0.40

^c^The normative data derived from the daily step count for special older populations suggested by Tudor-Locke et al. [42]

#### Test-retest reliability

Test-retest reliability was evaluated with 42 randomly selected participants at an interval of 7–14 days. All ICCs were strong to very strong (0.73–0.97), with the strongest reliability for the score of sitting (ICC = 0.97, 95% CI = 0.94–0.98). Strong reliability was also observed for the total PA score (ICC = 0.75, 95% CI = 0.58–0.86; SEM = 0.59). The result of the MDC was smaller than 10% for the total score, which may reflect a satisfactory parameter when comparing the mean between test and retest. The Wilcoxon signed-rank test indicated no significant difference (*p* > 0.05) for the retest at an interval of 7–14 days. The results of the Spearman rank correlation between test and retest indicated a moderate to strong correlation (*ρ* = 0.67–0.89, significant at the 0.01 level [two-tailed]). None of the 42 participants reported sports-activity-related information for calculating the test-retest reliability (Table 2). Additional analysis confirmed that there were no statistically significant differences in anthropometric characteristics between the 42 randomly selected participants and the total 124 participants (*p* > 0.05).

**Table 2:** Test-retest reliability of the APAFOP-C at intervals of 7–14 days *N* = 42

Variable	Test	Retest	Correlation coefficient		SEM	MDC_95_
			ICC/Spearman (95% CI)	Z (*p*)		
Total, S	28.46 ± 1.12	28.39 ± 1.09	0.73 (0.55–0.84) ^**#**^		0.59	1.62
Walking, RT	1.08 (0.67–1.60)	1.08 (0.73–1.56)	0.67 (0.38–0.86) ^¶^	−0.59 (0.557)		
Walking, S	2.16 (1.34–3.20)	2.16 (1.46–3.13)	0.67 (0.38–0.86) ^¶^	−0.59 (0.557)		
Outdoor activity, RT	0.33 (0.00–1.04)	0.38 (0.00–1.02)	0.85 (0.72–0.94) ^¶^	−0.17 (0.867)		
Outdoor activity, S	0.83 (0.00–2.12)	0.75 (0.00–2.04)	0.85 (0.70–0.94) ^¶^	−0.18 (0.861)		
Indoor activity, RT	4.64 ± 1.38	4.67 ± 1.63	0.78 (0.63–0.88) ^**#**^		0.65	1.80
Indoor activity, S	6.96 ± 2.06	7.01 ± 2.44	0.79 (0.63–0.88) ^**#**^		0.96	2.67
Sitting, RT	5.11 ± 2.51	5.07 ± 2.42	0.97 (0.94–0.98) ^**#**^		0.45	1.24
Sitting, S	5.11 ± 2.51	5.07 ± 2.42	0.97 (0.94–0.98) ^**#**^		0.45	1.24
Lying down, RT	11.33 (10.08–14.00)	11.66 (9.83–14.50)	0.89 (0.77–0.95) ^¶^	−0.10 (0.923)		
Lying down, S	11.33 (10.08–14.00)	11.66 (9.83–14.50)	0.89 (0.77–0.95) ^¶^	−0.16 (0.871)		
Sports, RT	-	-	-	-	-	-
Sports, S	-	-	-	-	-	-

^**#**^ ICC_2,1_; ^¶^ Spearman correlation coefficient; Z: Wilcoxon rank-sum test

Note. Data are mean ± SD or median (interquartile range) values; MDC_95_: minimal detectable change at the 95% CI

S: scores was calculated by duration × intensity; ICC: intraclass correlation coefficient; RT: real time measured (hour); SEM: standard error of the measurement

**Table 3:** Inter-rater reliability among three raters for APAFOP-C *N* = 124

Variable (S)	Correlation coefficient				
	ICC/Spearman (95% CI), *Z* (*p*)				Kendall’s W
	Rater 1^a^ vs. Rater 2	Rater 1^a^ vs. Rater 3		Rater 2 vs. Rater 3	
Total (S) ^¶^	0.89 (0.83–0.83), − 0.40 (0.690)		0.97 (0.93–0.99), − 1.86 (0.063)	0.88 (0.79–0.94), − 0.47 (0.639)	0.94**
Walking (S) ^¶^	0.95 (0.91–0.98), − 1.31 (0.189)		0.96 (0.93–0.98), − 0.34 (0.736)	0.94 (0.90–0.96), − 0.73 (0.471)	0.97**
Outdoor activity (S) ^¶^	0.97 (0.93–0.99), − 0.09 (0.927)		0.98 (0.97–0.99), − 1.27 (0.204)	0.95 (0.90–0.99), − 1.25 (0.210)	0.98**
Indoor activity (S) ^¶^	0.96 (0.94–0.98), − 0.30 (0.764)		0.95 (0.88–0.99), − 9.01 (0.061)	0.93 (0.85–0.98), − 9.15 (0.205)	0.97**
Sitting (S) ^#^	0.98 (0.97–0.98)		0.98 (0.97–0.99)	0.96 (0.94–0.97)	0.99**
Lying down(S) ^¶^	0.93 (0.84–0.98), − 2.49 (0.221)		0.90 (0.85–0.94), − 0.66 (0.507)	0.88 (0.81–0.94), − 0.76 (0.45)	0.94**
Sports (S) ^¶^	1.00		1.00	1.00	1.00**

^**#**^ ICC; ^¶^ Spearman; Z: Wilcoxon rank-sum test; ^a^ Reference rater

Note. ICC: intraclass correlation coefficient; S: score was calculated by duration × intensity

### Inter-rater reliability

Overall, under the null hypothesis that the ratings of the three raters are not concordant in Kendall’s W test, the total and each sub-domain PA scores between the three raters demonstrate absolute agreement (W = 0.94-1.00, *p* < 0.01). The Spearman rank correlation results also indicated a strong to very strong correlation (ρ = 0.88–0.97, 95% CI = 0.79–0.99, *p* < 0.01), and the Wilcoxon signed-rank test indicated that there was no significant difference (*p* > 0.05) among the total PA scores of the raters (Table 3). To be specific, compared with reference-rater 1 (median = 28.59, IQR = 27.62–29.92), rater 2 (median = 28.60, IQR = 27.59–29.63) and rater 3 (median = 28.61, IQR = 27.67–30.01) slightly overestimated the APAFOP-C-derived total PA scores of the participants. In addition, Rater 3 also slightly overestimated scores for indoor activities and lying down compared with rater 2, but there was still a very strong correlation among the scores of the three raters (indoor activities: ρ = 0.93–0.96, 95% CI = 0.85–0.99, *p* < 0.01; lying down: ρ = 0.88–0.93, 95% CI = 0.81–0.98, *p* < 0.01). The Wilcoxon signed-rank tests for each APAFOP-C domain results indicated that there was no significant difference between paired scores of the three raters (*p* > 0.05) (Table 3). Furthermore, very strong correlations were found among the scores of the raters for walking (ρ = 0.94–0.96, 95% CI = 0.90–0.98, *p* < 0.01), outdoor activity (ρ = 0.95– 0.98, 95% CI = 0.90– 0.99, *p* < 0.01), sitting (ICC = 0.96–0.98, 95% CI = 0.94–0.99, *p* < 0.01), and sports activity (ρ = 1.00, *p* < 0.01) (Table 3).

**Fig. 2:**
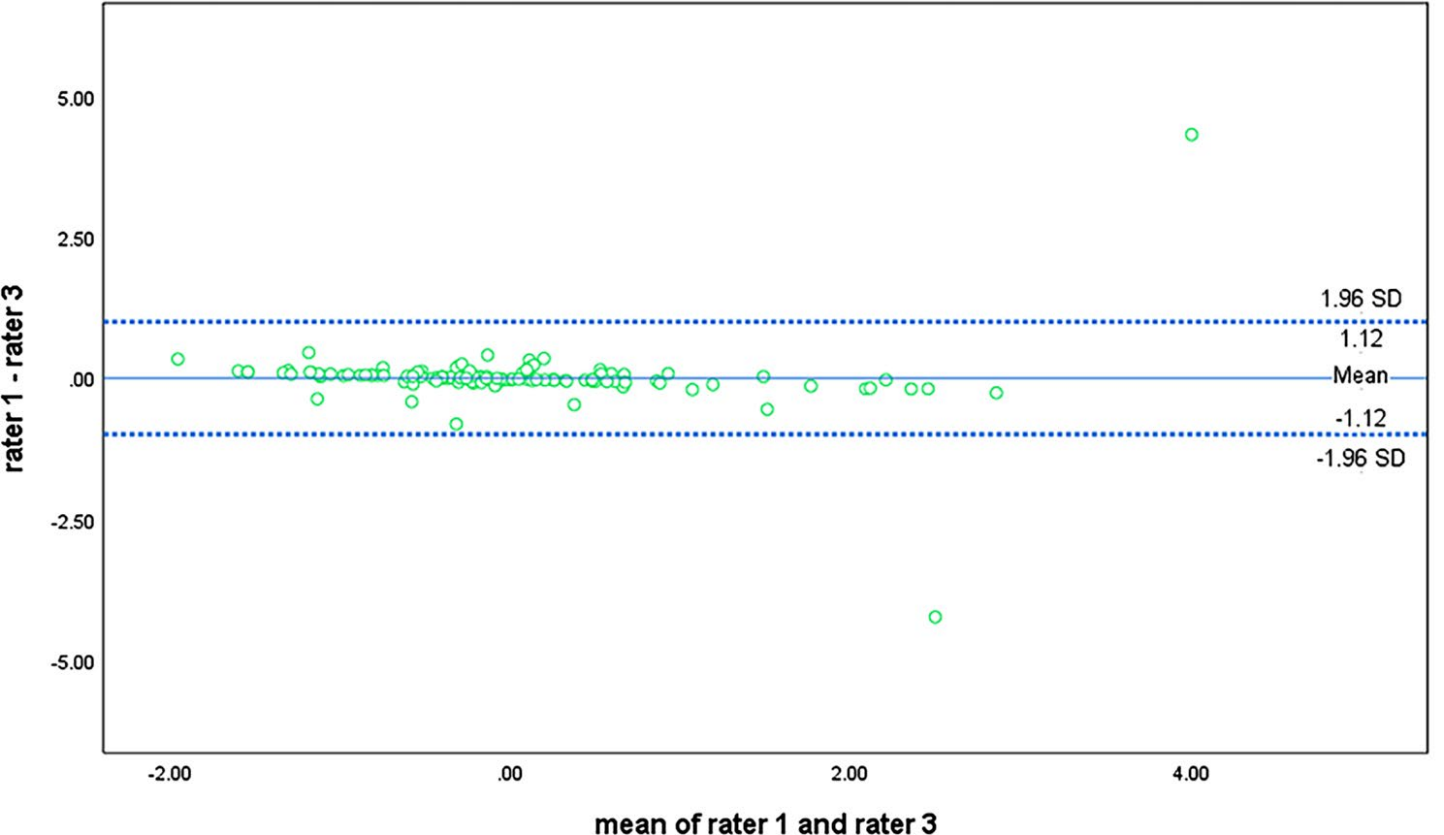
Bland-Altman plot of total PA score agreement between rater 1 and rater 3

**Fig. 3:**
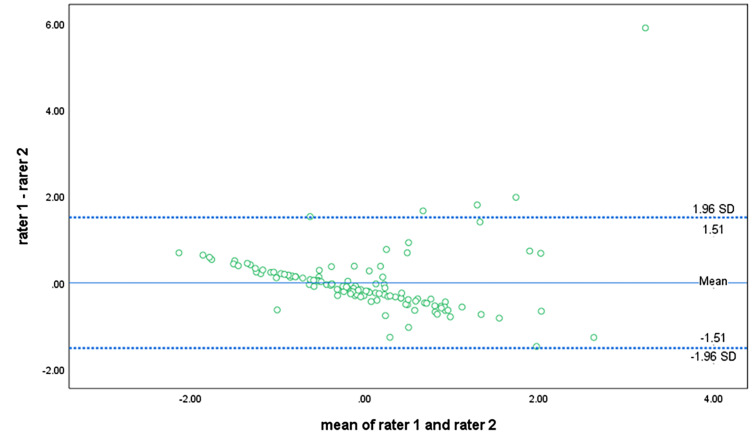
Bland-Altman plot of total PA score agreement between rater 1 and rater 2

**Fig. 4:**
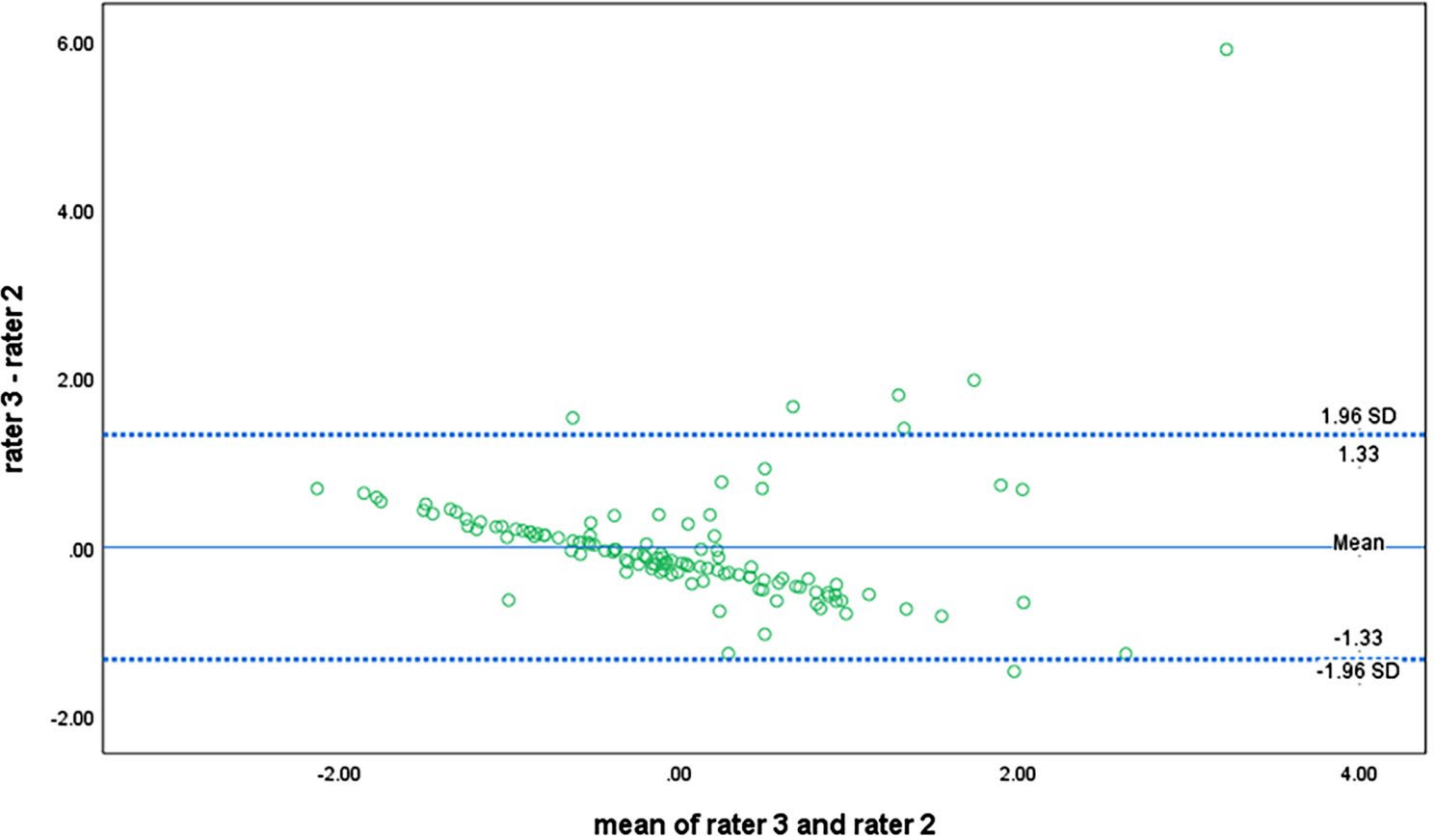
Bland-Altman plot of total PA score agreement between rater 3 and rater 2

The findings from the Bland-Altman plots indicated that the difference in limits of agreements (LoAs) between reference-rater 1 and any of the other raters obtained a threshold that marked a clear but acceptable difference. Following the plot for the agreement between rater 1 and rater 3 (Fig. 2), it appeared that a near-perfect correlation existed for the APAFOP-C but with slight bias, as most points within the plot were close to the mean and zero line. The LoAs for rater 1 and rater 3 were also narrow, with a difference in lying down of between − 1.12 and 1.12. Furthermore, both plots (Figs. 3 and 4) presented linear relationships that could be observed across the mean line, suggesting that rater 2 over- to under-reported total PA scores as the mean increased. These LoAs were also narrow, with differences between rater 1 and rater 2 of − 1.51 to 1.51 (Fig. 3) and between rater 3 and rater 2 of − 1.33 to 1.33 (Fig. 4).

### Criterion validity

**Fig. 5:**
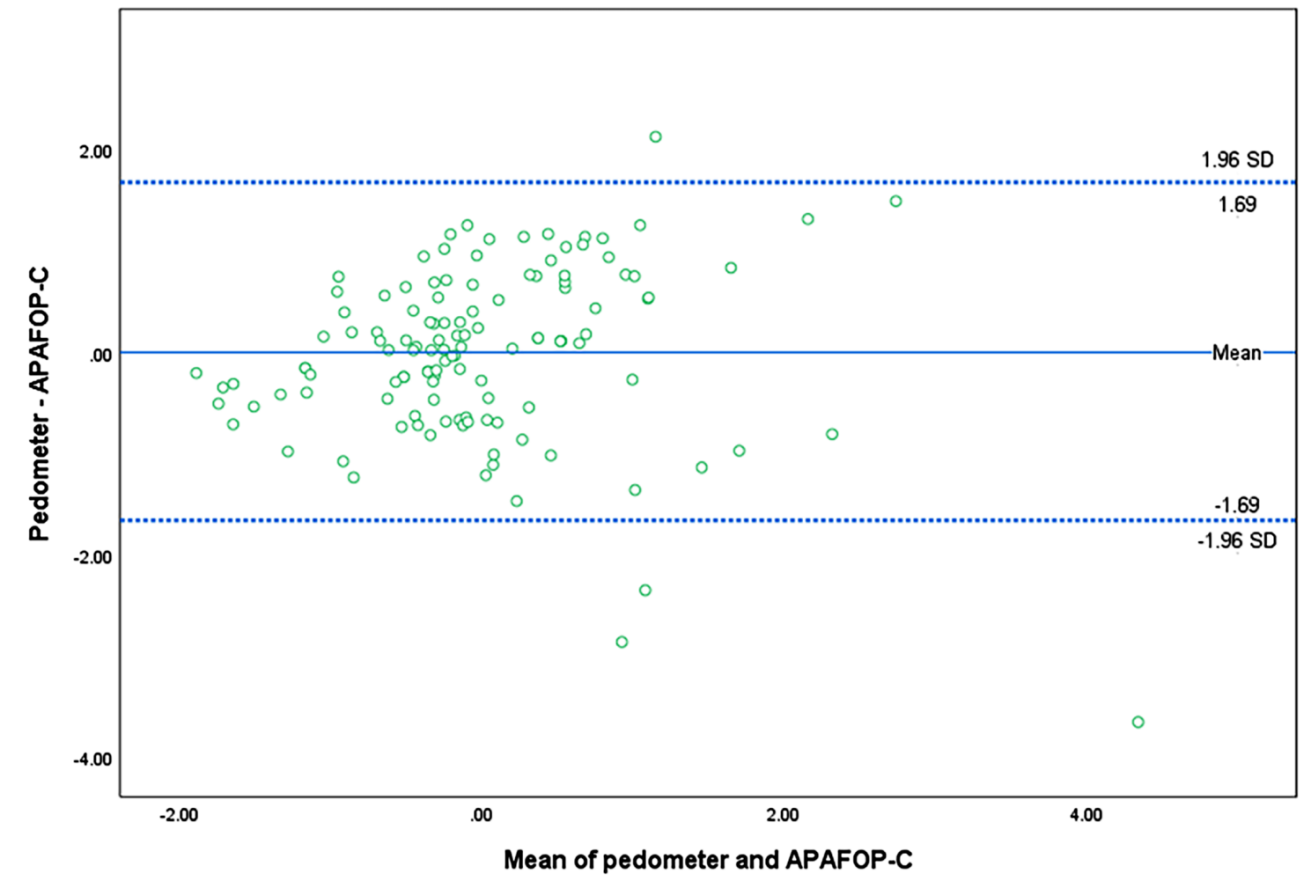
Bland-Altman plot of agreement of total PA score between the APAFOP-C and pedometer readings. *Note* The data in this Bland-Altman plot have been normalized to Z-scores, which adjust for differences in measurement scales to make them comparable. The *x*-axis represents the mean Z-score of the pedometer and APAFOP-C total score, while the *y*-axis shows the difference between the Z-scores of the pedometer readings and APAFOP-C total scores

Criterion validity was assessed with the data of 124 participants by comparing the scores of APAFOP-C with total step counts of the pedometer, an objective measure of PA selected as a gold standard. The total score of APAFOP-C along with the active or inactive PA scores calculated based on the domain activities were used for the comparisons. The results showed moderate correlations for inactive PA (ρ=−0.58, 95% CI = − 0.45- −0.071), active PA (ρ = 0.60, 95% CI = 0.45–0.71), and total PA scores (*ρ* = 0.61, 95% CI = 0.46–0.72) with the pedometer readings. Weak but significant correlations were observed for walking (ρ = 0.37, 95% CI = 0.20–0.53) and indoor activity (ρ = 0.32, 95% CI = 0.14–0.48). A negative but significant correlation was found for the sitting score (*r* = − 0.27, *p* < 0.01). No significant correlations were found in the outdoor activity, lying down, and sports activity domains with the pedometer readings (Table 4). In addition, the results of the Bland-Altman analysis for total PA score and pedometer readings demonstrated a narrow LoA (Fig. 5).

**Table 4:** Criterion validity of APAFOP-C with pedometer readings

APAFOP domains		*N* = 124 Correlation coefficient (95% CI)
Total PA score		0.61 (0.46–0.72) ^¶^
Active PA score		0.60 (0.45–0.71) ^¶^
	Walking (S)	0.37 (0.20–0.53) ^¶^
	Outdoor activity (S)	0.15 (− 0.03 to 0.31) ^¶^
	Indoor activity (S)	0.32 (0.14–0.48) ^¶^
	Sports activity (S)	0.10 (0.08–0.20) ^¶^
Inactive PA score		−0.58 (− 0.45 to − 0.71) ^¥^
	Sitting (S)	−0.27 (− 0.08 to − 0.45) ^¥^
	Lying down (S)	−0.12 (− 0.30 to 0.09) ^¶^

^¶^ Spearman correlation coefficient; ^**¥**^ Pearson correlation coefficient

*Note* S: score was calculated by duration × intensity; Total PA score: summed scores of all domains; Active PA score: summed scores of walking, outdoor activities, indoor activities, and sports activities; Inactive PA score: summed scores of sitting and lying down

## Discussion

This study assessed the test-retest, inter-rater, and criterion validity of the Chinese version of APAFOP among frail older adults living in the community. The results indicated acceptable test-retest and inter-rater reliability for the total PA scores, as well as the subdomain scores of APAFOP-C. An objective measure of physical activity, the pedometer, showed moderate criterion validity when compared with the total score, inactive PA, and active PA scores of the APAFOP-C.

This study supports the test-retest reliability of the APAFOP-C at intervals of 7–14 days, with a moderate-to-very strong correlation. This result was consistent with those of Hauer et al. [23], who found no significant variation in the total PA score among both cognitively impaired (ICC = 0.98) and normal (ICC = 0.97) older adults with frailty. Moreover, our results were comparable to those of another study testing the psychometric properties of the APAFOP, which demonstrated the highest test–retest reliability (ICC = 0.99) [24].

We also evaluated inter-rater reliability using Bland-Altman plots. The calculations in this study were based on absolute agreement among three raters and pair-wise comparisons of different raters for each participant. The results indicated that the correlation coefficient values were all strong to very strong, and the Bland-Altman plots indicated that the differences in LoA between reference-rater 1 and the other two raters were clearly different. However, each comparison showed a narrow LoA. In comparison with the mean line, the variation was smallest between reference-rater 1 and rater 3, who received formal training before the study. In comparison to other raters, Rater 2 overestimated total PA scores, but the difference was not statistically significant, and the LoA was narrow. The results support the hypothesis that prior training can minimize inter-rater variability, while utilizing user manuals without formal training also results in reliable results.

When total APAFOP-C score was compared with a pedometer as a gold standard measure of PA, there was a moderate correlation in criterion validity. The Bland-Altman analysis also indicated good agreement between total APAFOP-C score and pedometer readings. These results were comparable to those of Hauer et al. [23] and Moldes et al. [24], who demonstrated correlations with Physilog- and accelerometer-derived data as *r* = 0.70 and *r* = 0.65, respectively, and found a narrow LoA (from − 3.163 to 3.775) [23]. However, the correlations of pedometer readings were not sufficient as a gold standard when comparing active and inactive PA scores of APAFOP-C [43]. Pedometers tend to underestimate steps in older adults who live in the community [44], especially those with slower gait speeds. In motion-capture systems like pedometers, which measure vertical accelerations to define positions, it would be difficult to distinguish between lying down and sitting, particularly when frail older adults rest in almost-lying-down positions [45]. In spite of these limitations, good agreement by the Bland-Altman analysis and significant negative correlation between pedometer steps with inactive PA scores (such as lying down and sitting) suggests that the APAFOP-C is sensitive to capturing activities involving minimal physical movement. For frail older individuals, these low-intensity activities and inactive postures occupy most of their time, so the APAFOP-C can be used to assess their physical activity profiles.

When we compared inactive APAFOP-C scores (outdoor and sports activity) with pedometer readings, we found weaker correlations, probably because only a few older adults reported doing these types of activities. Both of these activities require older adults with frailty to leave their homes, and many of them do not enjoy exercising. Furthermore, the data were collected during the COVID-19 pandemic, which significantly decreased outdoor or sports-related activity time for older adults with frailty [46]. According to these findings, it is warranted to examine the criterion validity of APAFOP-C in relation to these types of physical activities.

The study has several strengths, including the fact that we are focusing on physical activities of frail older adults in the community, a group that is typically understudied and neglected. In contrast to most PA questionnaires, the APAFOP-C required participants to recall their PA over the previous 24 h, allowing for the detection of subtle changes in PA over time. The strong inter-rater reliability demonstrated suggests that the APAFOP-C user manual was clear and beneficial for researchers, and it was confirmed that adherence to the guidelines provided in the manual during the interviews could effectively minimize measurement bias.

However, several limitations should be considered in the interpretation of the findings. Due to the fact that the pedometer provides only total steps for 24 h and was insensitive to inactive physical activity, the criterion validity of certain subdomains of APAFOP-C was not sufficient to the required level. The environmental factors (COVID-19 pandemic) during the data collection period may also contribute to the reduced outdoor or sports activity participation for this population, which made more difficult to assess the full range of activities by both objective and subjective measures. In addition, due to the interview-based nature and the subjectivity of the APAFOP-C, there is potential for measurement error compared to the recording-based measure. We recommend that researchers thoroughly review the user manual prior to conducting interviews to minimize various biases. Although we demonstrated that the APAFOP-C is a valid and cost-effective measure of PA in older adults with frailty, there are unique challenges in applying this scale. While PA variability appears to decrease with age and limited functional status, PA is not a static behavior and involves multiple separate dimensions. Our findings based on test-retest reliability provided ample evidence that the APAFOP-C can record the habitual PA of the participants. However, this daily variation of PA in frail and sedentary populations cannot be ignored. It is recommended that future studies examine whether the APAFOP-C could effectively assess these subtle changes in PA since PA can provide a roadmap for treating and preventing frailty in this population [8].

## Conclusion

The findings of this study suggest that the APAFOP-C is a feasible PROM with reasonable psychometric properties and is reliable in assessing different intensities and various domains of PA among community-residing older adults with frailty. The APAFOP-C provides a tailored approach to assess the PA level of older adults with frailty over a relatively short period. Moreover, the study has highlighted the need to use the same questionnaire in surveillance studies to compare and follow up on the PA levels of older adults with frailty and to develop individualized exercise programs based on the data derived from the APAFOP-C. The APAFOP-C also retains the same calculation and classification of items as the original English version; only some activities were modified due to the characteristics of the Chinese circumstances, and the layout was changed for convenience. The APAFOP-C therefore seems to be an efficient and low-burden assessment tool that can be used to determine and differentiate PA levels in frail older adults.

### Electronic supplementary material

Below is the link to the electronic supplementary material.

Supplementary Material 1

## Data Availability

The datasets generated and/or analysed during the current study are available in the ICPSR repository (openicpsr-198486).

## Abbreviations

PA: Physical activity
APAFOP: Assessment of physical activity in frail older people
APAFOP-C: Chinese version of the assessment of physical activity in frail older people
ICC: Intraclass correlation coefficient
MDC_95_: Minimal detectable changes at the 95% confidence level
SEM: Standard error of measurement
PROM: Patient-reported outcome measure
LoA: Limit of agreement
