# Supplementary material for: Reliability, validity and minimal detectable change of the Chinese Version of the Assessment of Physical Activity in Frail Older People (APAFOP-C)
Source: BMC Geriatr. 2024 Jul 6;24:582. doi: 10.1186/s12877-024-05167-y (PMC11227165; doi:10.1186/s12877-024-05167-y)
Supplement: Supplementary file 1 — Supplementary Material 1 [file 12877_2024_5167_MOESM1_ESM.pdf]

APAFOP-C 使用手册

(Assessment of Physical Activity in Frail Older People-Chinese version)

1. 问卷介绍

本问卷由两部分组成，第一部分附录表 1 即问卷条目，不同类型活动和对应 MET 分数；第二部分是评估表即附录表 2 日常活动评估表（包括条目 1 步行，2 户外活动，3 室内活动，4 坐着，5 躺着）和附录表 3 体育活动评估表（条目 6 体育活动）

2. 半结构化访谈提纲

2.1 开场语

您好，我对您的这次访谈是为了对您昨天的活动进行记录。为了后期的准确记录，本次访谈会进行录音，录音仅用于后期核对，绝对保密。访谈时间可能在 8 分钟左右，请问您是否愿意接受这次访谈呢？好的，那我们开始吧！

2.2 访谈问题及示例

采访者提问	受访者回答	记录[时间、活动（强度）、小时、MET]
1.请问您昨天是几点起床的？	早上 6 点起床的。	0:00-6:00，躺着，6hr，1
2.起床后做了什么呢？比如听广播，看电视，洗漱，做早餐，请精确到分钟。	6 点起床后，躺着听广播到 6:30，然后洗漱到 6:40，准备了早饭，吃完早饭大概是 7 点。	6:00-6:30，躺着，0.5hr，1<br>6:30-6:40，室内（低），0.17hr，1.5<br>6:40-7:00，室内（低），0.33hr，1.5
3.吃完早餐之后做了什么呢？请努力回忆并精确到分钟。	吃完早饭是 7 点，之后和孙女打了电话到 7：20 结束，然后就一直在看电视织毛衣到 11：00。	7:00-7:20，室内（低），0.33hr，1.5<br>7:20-11:00，坐着，3.67hr，1
4.那 11 点织完毛衣之后做了什么呢？请努力回忆并精确到分钟。	织完毛衣后，去上了卫生间，大概是 11:05 完事，然后去准备午饭，吃完午饭是 11:40，躺着休息了一会，大概 12:30 起来的。	11:00-11:05，室内（低），0.08hr，1.5<br>11:05-11:40，室内（低），0.58hr，1.5<br>11:40-12：30，躺着，0.83hr，1
5.好的，那您午休后做什么了？请努力回忆并精确到分钟。	午休后，简单收拾了一下屋子，然后准备去社区和朋友见面，大概是 13:00 出发，到那里大概是 13:20，一直在聊天，15:00 的时候我们有健身操，15:30 结束，然后就回家了，到家大概是 16:00。	12:30-13:00，室内（低），0.5hr，1.5<br>13:00-13:20，步行，0.33hr，2<br>13:20-15:00，室内（低），1.67hr，1.5<br>15:00-15:30，体育（高），0.5hr，4<br>15:30-16:00，步行，0.5hr，2
6.到家之后做了什么呢？请努力回忆并精确到分钟。	准备做饭，吃饭，洗碗，大概 16:30 结束，然后一直看电视到 21:00，然后洗漱到 21:30，然后上床睡觉了。	16:00-16:30，室内（低），0.5hr，1.5<br>16:30-21:00，坐着，4.5hr，1<br>21:00-21:30，室内（低），0.5hr，1.5<br>21:30-24:00，躺着，2.5hr，1

3. 有助于准确报告和增加受访者回忆召回的方法

3.1 判断步行的时间

时间超过 3 分钟的步行涉及条目 1，时间小于 3 分钟的步行根据环境分类，在家附近，街道，购物等属于条目 2“户外活动”；绕客厅走，在屋子里边走边打电话属于条目 3“室内活动”。

3.2 谨防系统误差

本问卷属于高度结构化访谈问卷，6 项条目时间之和必须等于 24 小时，且已规定分钟和小时的换算单位，见附录表 2-*便捷转换*（访谈时以 5，10，15…分钟为单位记录，以便换算）。

3.3 记录晚间睡眠时长

例如 2.2 示例中受访者说 6 点起床，则记录“0:00-6:00，躺着，6hr，1”，受访者说 21:30 上床睡觉则记录“21:30-24:00,躺着，2.5hr，1”。

3.4 增加受访者回忆召回的方法

3.4.1 采用非正式的对话方式。

3.4.2 使用特定的时间范围作为“分割点”，将过去 24 小时记忆分为线索和结构记忆（见附录表 2- 起床、早餐、午餐、晚饭、睡觉）。

APAFOP-C 条目、活动权重、评价表

附录表 1：问卷条目、不同类型活动及强度评分

条目	强度分类	不同强度对应的活动	METs 分数
1. 步行 ※（超过 3 分钟）	低	散步，步行到商店或市场，送孩子上学，遛狗	2
	中	把走路当锻炼	3
	高	爬坡或快步走	4
2. 户外活动	低	家附近（在住宅楼附近散步，在花园浇水，在花园散步）	2
		离家远（扫墓，郊游，野餐，早市买菜）	
	中	打扫院子或街道，购物等，各种活动期间步行比例的增加	3
	高	更费力的园艺活动，维修摩托车等机械操作	4
3. 室内活动	低	轻松的家务（洗碗，做饭，吃饭，浇花，被褥整理）<br>个人卫生（洗漱，沐浴，上厕所）	1.5
		休闲社会活动（站立时/边走边打电话，串门，与邻居交谈，打麻将，下棋，练书法）	
		其他（照顾宠物，绕客厅走，混合活动但着重于站立）	
	中	劳累的家务（洗衣服，整理购买的物品，吸尘，拖地，打扫房间）	2
		混合活动但着重于步行	
	高	搬运及整理重物（米袋，面袋，箱柜），爬楼梯	3
		为有生活依赖者（儿童，瘫痪，脑瘫等患者）提供特别护理或帮助	
4. 坐着		坐着看电视，打电话，交谈，乘坐交通工具时坐着，在餐厅或剧场坐着	1
5. 躺着		躺着读书，看电视，睡觉	1
6. 体育活动	低	坐着或躺着进行伸展运动，非剧烈混合运动	2
	中	中等耐力的家庭训练,站立或步行时的中等运动（倒退步行，侧向走，脚跟走，脚趾走）	3
	高	无氧运动（使用哑铃，举重机，俯卧撑，手持式重物进行的锻炼）	4
		有氧运动（瑜伽、太极拳、健美操）	
		强度更高的耐力运动（如骑自行车，网球，羽毛球，足球，游泳，跑步/慢跑，广场舞）	
		其他具有挑战性的运动	

MET= metabolic equivalent 代谢总量.

附录表 2：老年人日常活动评估表

过去 24 小时的活动类型	记录区域	步行 [hr] (MET)	户外 [hr] (MET)	室内 [hr] (MET)	坐着 [hr] (MET)	躺着 [hr] (MET)	体育活动 [hr] (MET)
**例**	0:00-6:00 睡觉(躺着)					6hr(1)	
	6:00-6:30 洗漱(室内;低)			0.5hr (1.5)			
▷起床<br>▷早餐<br>▷午餐<br>▷晚餐<br>▷睡觉							
持续总时间（竖向相加）=hr							
分数（MET 与相应运动时间相乘）<br>= MET× hr		2×__=<br>3×__=<br>4×__=	2×__=<br>3×__=<br>4×__=	1.5×___=<br>2×___=<br>3×___=	1×___=	1×___=	2×__=<br>3×__=<br>4×__=

便捷转换:
☆5min=0.08hr ☆10min=0.17hr ☆15min=0.25hr ☆20min=0.33hr
☆25min=0.42hr ☆30min=0.5hr ☆35min=0.58hr ☆40min=0.67hr
☆45min=0.75hr ☆50min=0.83hr

附录表 3：老年人活动评估总分

活动类型（各项相加）	时间（hr）<br>※（6 条目时间之和必须等于 24hr）	分数
1. 步行		
2. 户外活动		
3. 室内活动		
4. 坐着		
5. 躺着		
6. 体育运动		
总指数		
